# Presentation patterns and management outcomes of uterine fibroids: A retrospective study

**DOI:** 10.6026/973206300213986

**Published:** 2025-10-31

**Authors:** Neha Kumari, Sadhana Singh, Kamlesh Tiwary

**Affiliations:** 1 Department of Obstetrics and Gynaecology, Radha Devi Jageshwari Memorial Medical College and Hospital, Dr. Kalam Nagar, Manariya Chhajan, Turki, Muzaffarpur, Bihar, India

**Keywords:** Uterine fibroids, leiomyoma, myomectomy, hysterectomy, heavy menstrual bleeding, retrospective study

## Abstract

Uterine fibroids are common benign pelvic tumors in reproductive-age women, often presenting with heavy menstrual bleeding and pelvic pain. Therefore, it is of interest to analyze 60 patients undergoing myomectomy (66.7%) or hysterectomy (33.3%) between 2022 and 2024. Intramural fibroids (60.0%) with a mean size of 8.2 cm were the most frequent finding. Symptom resolution at 6 months was higher after hysterectomy (95.0%) compared to myomectomy (82.5%, p=0.048), while complication rates were similar. Surgical management provides effective relief, with hysterectomy offering a definitive cure and myomectomy serving as a fertility-preserving option.

## Background:

Uterine leiomyomas, commonly known as fibroids, are benign monoclonal tumors arising from the smooth muscle cells of the myometrium. They represent the most prevalent pelvic neoplasm in premenopausal women, with an estimated cumulative incidence of over 70% by the age of 50 [1]. While many fibroids remain asymptomatic, approximately 20-50% of affected women experience significant symptoms that can profoundly impair their physical, social and emotional well-being [2]. The clinical presentation of uterine fibroids is highly variable and depends on their number, size and location within the uterus. Common symptoms include heavy menstrual bleeding (HMB), which can lead to anemia; chronic pelvic pain or pressure; dysmenorrhea; and bulk-related symptoms such as urinary frequency and constipation [3]. Furthermore, fibroids, particularly those that distort the uterine cavity submucosal or are intramural, have been implicated in reproductive dysfunction, including infertility, recurrent pregnancy loss and adverse obstetric outcomes [4]. The management of symptomatic fibroids is tailored to the individual, considering factors such as symptom severity, patient age, desire for future fertility and proximity to menopause. Treatment modalities range from expectant management and medical therapies (e.g., hormonal contraceptives, gonadotropin-releasing hormone agonists) to more definitive procedural interventions [5]. Surgical options remain the mainstay for patients with severe symptoms or those who have failed medical management. Myomectomy, the surgical removal of fibroids while preserving the uterus, is the preferred option for women who wish to retain their fertility. Hysterectomy, the complete removal of the uterus, provides a definitive cure for fibroid-related symptoms and is typically reserved for women who have completed childbearing [6]. Numerous studies have investigated the epidemiology and clinical impact of uterine fibroids globally. However, presentation patterns and management choices can vary across different geographical and socio-economic populations due to genetic predispositions, healthcare access and cultural factors [7]. While the fundamental pathophysiology is understood, a significant research gap exists in the form of updated, institution-specific data that reflects current clinical practice and outcomes. Such localized data is essential for auditing clinical services, refining treatment protocols and providing accurate patient counseling. Therefore, it is of interest to evaluate the clinicopathological presentation patterns and surgical management outcomes of uterine fibroids in patients treated at our tertiary care institution.

## Materials and Methods:

## Study design and setting:

This was a retrospective, descriptive, record-based Study at the Department of Obstetrics and Gynaecology, Radha Devi Jageshwari Memorial Medical College and Hospital, Dr. Kalam Nagar, Manariya Chhajan, Turki, Muzaffarpur, Bihar, India.

## Study population and period:

The study included the medical records of all patients who underwent surgical intervention (myomectomy or hysterectomy) for a primary diagnosis of uterine fibroids between two years. A total of 60 patient records that met the inclusion criteria were selected for the final analysis through a non-probability, purposive sampling technique.

## Inclusion and exclusion criteria:

## Inclusion criteria were:

(1) women aged between 18 and 50 years; (2) a pre-operative diagnosis of uterine fibroids confirmed by transvaginal or transabdominal ultrasonography; (3) surgical management via abdominal myomectomy or total abdominal hysterectomy; and (4) a postoperative histopathological confirmation of leiomyoma.

## Exclusion criteria included:

(1) patients with a co-existing diagnosis of adenomyosis, endometriosis, or pelvic inflammatory disease that was the primary indication for surgery; (2) suspected or confirmed uterine or cervical malignancy; (3) management with minimally invasive techniques such as laparoscopy, hysteroscopy, or uterine artery embolization; and (4) medical records with incomplete clinical or follow-up data.

## Data collection procedure:

A pre-designed, structured data collection proforma was used to extract information from the hospital's electronic health records and physical case files.

## The following variables were collected:

[1] Demographic data: Age at surgery, parity.

[2] Clinical presentation: Chief complaints (heavy menstrual bleeding, dysmenorrhea, pelvic pain/pressure, infertility, palpable abdominal mass).

[3] Diagnostic findings: Pre-operative hemoglobin levels, findings from pelvic ultrasonography, including the number of fibroids (single, multiple), location (subserosal, intramural, submucosal) and the size of the dominant fibroid.

[4] Surgical and postoperative data: Type of surgical procedure performed (myomectomy or hysterectomy), intraoperative findings, postoperative histopathology report, duration of hospital stay, occurrence of any immediate postoperative complications (e.g., hemorrhage, fever, wound infection) and symptom status at the 6-month follow-up visit.

## Statistical analysis:

The collected data were coded and entered into a spreadsheet (Microsoft Excel 2019) and subsequently analyzed using the Statistical Package for the Social Sciences (SPSS) version 26.0. Descriptive statistics were calculated for all variables. Continuous data (e.g., age, fibroid size) were expressed as mean ± standard deviation (SD), while categorical data (e.g., symptoms, fibroid location) were presented as frequencies and percentages (%). The Student's t-test was used to compare the means of continuous variables between the myomectomy and hysterectomy groups. The Chi-square (χ^2^) test or Fisher's exact test, where appropriate, was used to compare categorical variables. A p-value of less than 0.05 was considered to be statistically significant.

## Results:

A total of 60 patients who underwent surgery for uterine fibroids were included in the analysis. The baseline demographic and clinical presentation details are summarized in Table 1 (see PDF). The mean age of the study population was 38.5 ± 6.2 years, with an age range of 18 to 50 years. The majority of patients were multiparous (63.3%). The most common presenting symptom was heavy menstrual bleeding (HMB), reported by 45 patients (75.0%). This was followed by pelvic pain or pressure symptoms, which were present in 32 patients (53.3%). A significant proportion of women also reported dysmenorrhea (46.7%). Primary or secondary infertility was the chief complaint in 10 patients (16.7%). The ultrasonographic and histopathological characteristics of the fibroids are shown in Table 2 (see PDF). Multiple fibroids were more common than single fibroids, found in 40 patients (66.7%). The mean size of the dominant fibroid was 8.2 ± 3.1 cm. In terms of location, intramural fibroids were the most frequent type, identified in 36 patients (60.0%) and followed by subserosal fibroids (33.3%). Surgical management consisted of myomectomy for 40 patients (66.7%) and hysterectomy for 20 patients (33.3%). A comparison of the two surgical groups is presented in Table 3 (see PDF). There was a statistically significant difference in the mean age between the two groups, with patients undergoing myomectomy being considerably younger than those undergoing hysterectomy (35.1 ± 4.8 vs. 45.3 ± 5.1 years, p < 0.001). At the 6-month postoperative follow-up, complete symptom resolution was achieved in 33 of 40 (82.5%) myomectomy patients and 19 of 20 (95.0%) hysterectomy patients. This difference was found to be on the border of statistical significance (p=0.048). The overall rate of short-term postoperative complications was 13.3% (8 out of 60 patients). There was no significant difference in the complication rates between the myomectomy and hysterectomy groups (12.5% vs. 15.0%, p=0.812).

## Discussion:

This retrospective study provides a snapshot of the clinical profile and surgical management outcomes of uterine fibroids at our institution. The findings confirm that fibroids are a significant cause of gynecological morbidity, predominantly affecting women in their late reproductive years, with a mean age of 38.5 years in our cohort. This demographic is consistent with reports from other studies, which place the peak incidence in the fourth and fifth decades of life [8]. The most prevalent symptom identified in our study was heavy menstrual bleeding (75.0%), a finding that aligns with the majority of the existing literature [2, 7]. The high frequency of HMB is mechanistically linked to the presence of intramural and submucosal fibroids, which were the most common types in our cohort. These fibroids can increase the endometrial surface area, cause vascular disruption and impair normal uterine contractility, leading to excessive bleeding [9]. The significant proportion of patients with anemia (41.7%) secondary to HMB underscores the substantial impact of this symptom on women's health. The choice of surgical management in our study was clearly delineated by patient age and, by extension, the desire for fertility preservation. Patients who underwent myomectomy were, on average, a decade younger and more likely to be nulliparous compared to those who opted for hysterectomy. This reflects appropriate clinical decision-making, where uterus-sparing surgery is prioritized for women who have not completed their families, while hysterectomy is offered as a definitive treatment for older women with severe symptoms [6, 10]. In terms of outcomes, both surgical procedures were highly effective in providing symptom relief. The rate of complete symptom resolution was slightly higher in the hysterectomy group (95.0%) compared to the myomectomy group (82.5%). This is an expected outcome, as hysterectomy eliminates the source of the symptoms and precludes any possibility of fibroid recurrence, thereby offering a definitive cure [11, 12-13]. Similarly, Madunatu *et al.* in 2024 documented that intramural fibroids were the most common subtype and that hysterectomy resulted in higher symptom resolution compared to myomectomy in a 5-year Nigerian cohort, which closely aligns with the findings of the present study [14]. The small number of patients in the myomectomy group who did not experience complete resolution may have had residual small fibroids, co-existing adenomyosis, or other contributing factors. Importantly, our study found no statistically significant difference in the rates of short-term postoperative complications between abdominal myomectomy and hysterectomy. This suggests that both procedures, when performed in a tertiary care setting by experienced surgeons, carry a comparable and acceptable level of surgical risk. This finding is crucial for patient counseling, as it allows clinicians to reassure women opting for myomectomy that the procedure is not inherently more dangerous than a hysterectomy in the immediate postoperative period [15, 16, 17-18]. This study is subject to several limitations inherent to its design. The retrospective nature introduces the possibility of information bias and reliance on the accuracy and completeness of medical records. The small sample size of 60 patients limits the statistical power of our comparative analyses and the generalizability of our findings. Furthermore, this study only captured short-term outcomes at 6 months; it does not provide data on long-term fibroid recurrence rates after myomectomy or long-term quality of life improvements, which are critical aspects of fibroid management.

## Conclusion:

Uterine fibroids commonly present with heavy menstrual bleeding and bulk-related symptoms in women in their late 30s and 40s. The findings of this study confirm that surgical management, tailored to the patient's age and reproductive goals, is a highly effective treatment strategy. Myomectomy serves as a safe and effective uterus-preserving option for younger women, while hysterectomy provides a definitive cure for those who have completed childbearing, with both procedures demonstrating comparable short-term safety profiles. This local data reinforces established clinical guidelines and highlights the importance of individualized patient counseling to achieve optimal outcomes in the management of symptomatic uterine fibroids.
